# Prognostic and clinicopathological value of the geriatric nutritional risk index in gastric cancer: A meta-analysis of 5,834 patients

**DOI:** 10.3389/fsurg.2022.1087298

**Published:** 2023-01-06

**Authors:** Liang He, Ying Li, Linlin Qu, Fan Zhang

**Affiliations:** ^1^ Department of Gastrointestinal Surgery, The First Hospital of Jilin University, Changchun, China; ^2^ Department of Gastroenterology, The First Hospital of Jilin University, Changchun, China; ^3^ Department of Clinical Laboratory, The First Hospital of Jilin University, Changchun, China

**Keywords:** gastric cancer, prognosis, meta-analysis, risk factors, geriatric nutritional risk index

## Abstract

**Background:**

Recent studies have explored the prognostic value of the geriatric nutritional risk index (GNRI) in patients with gastric cancer (GC), but the results are controversial. We aimed to systemically identify the association between the GNRI and prognosis in GC using a meta-analysis.

**Methods:**

The databases of PubMed, Web of Science, Cochrane Library, and Embase were searched until September 25, 2022. Pooled hazard ratios and the corresponding 95% confidence intervals (CIs) were used to estimate the prognostic value of the GNRI in GC. Odds ratios (ORs) and 95% CIs were used to assess the correlation between the GNRI and clinicopathological characteristics of GC.

**Results:**

Ten studies including 5,834 patients with GC were included in this meta-analysis. The merged results indicated that a low pretreatment GNRI was associated with inferior overall survival (hazard ratio = 1.21, 95% CI = 1.12–1.30, *P* < 0.001) and worse cancer-specific survival (hazard ratio = 2.21, 95% CI = 1.75–2.80, *P* < 0.001) for GC. Moreover, a low GNRI was significantly associated with an advanced pathological stage (OR = 2.27, 95% CI = 1.33–3.85, *P* = 0.003), presence of adjuvant chemotherapy (OR = 1.25, 95% CI = 1.01–1.55, *P* = 0.040), and tumor location in the lower stomach (OR = 1.33, 95% CI = 1.06–1.65, *P* = 0.012) in GC. However, there was no significant association between GNRI and sex, tumor differentiation, or lymph node metastasis in patients with GC.

**Conclusion:**

Our meta-analysis identified that the pretreatment GNRI level was a significant prognostic factor for patients with GC. A low GNRI is associated with worse overall survival and inferior cancer-specific survival in patients with GC.

## Introduction

Gastric cancer (GC) is the fifth most prevalent cancer and fourth leading cause of cancer-related death worldwide (1). GC accounts for 5.6% of new cancer cases and 7.7% of cancer-related deaths in 2020 globally (1). Although its incidence and mortality have declined over the past several decades, more than one million cases of GC are diagnosed each year worldwide (2). Surgical resection is the mainstay of treatment for early GC, whose 5-year survival rate is approximately 80% (3). However, approximately 60% of patients with GC present with a late-stage diagnosis (4). The mortality rate of GC remains high, with 5-year survival rates ranging from 28% to 51% worldwide (5). Reliable prognostic markers could have important implications for the management of patients with GC. Therefore, identifying novel biomarkers is pivotal for the early prediction of prognosis so as to develop individualized treatment strategies for patients with GC.

Nutritional status is an important factor affecting the response and prognosis of patients with cancer, and approximately 30%–40% of patients have malnutrition (6). Previous studies have demonstrated the prognostic value of nutritional indexes in patients with GC, including the prognostic nutritional index (7), controlling nutritional status score (8), albumin-to-globulin ratio (9), and C-reactive protein to albumin ratio (10). The geriatric nutritional risk index (GNRI) is a novel nutrition-based parameter calculated as1.489 × albumin (g/dl) + 41.7 × actual body weight/ideal body weight (kg). It is favored to assess nutritional status and disease prognosis in older patients (11, 12). Previous studies have explored the prognostic significance of the GNRI in patients with GC; however, the results were inconsistent (13–22). For example, some studies have confirmed the independent prognostic role of the GNRI for survival in GC (13, 14, 16). However, other researchers have reported that the association between the GNRI and prognosis in GC was not significant (15, 21). Therefore, in this study, we retrieved the most recent data and performed a comprehensive meta-analysis to quantitatively identify the prognostic value of the GNRI in GC. Moreover, the association between the GNRI and clinicopathological features of GC was also investigated.

## Materials and methods

### Data sources and search strategy

This meta-analysis was conducted in accordance with the Preferred Reporting Items for Systematic Reviews and Meta-Analyses (PRISMA) statement (23); the checklist is provided in Supplementary Material S1. The databases of PubMed, Web of Science, Cochrane Library, and Embase were searched with the following search items: (geriatric nutritional risk index OR GNRI) AND (gastric cancer OR gastric carcinoma OR gastric adenocarcinoma OR stomach neoplasm OR stomach cancer). Data were collected from the inception of each database to September 25, 2022. The language of the studies was limited to English. Additionally, the reference lists of relevant studies were manually reviewed to identify additional studies.

### Inclusion and exclusion criteria

The inclusion criteria for this study were formulated on the basis of the Population-Intervention-Control-Outcome (PICO) framework.

The inclusion criteria were as follows: (1) P (patients): patients who were pathologically diagnosed with primary GC; (2) I (intervention—exposure): patients with malnutrition risk as determined by a low pretreatment GNRI level; (3) C (control): patients with a normal nutritional status as determined by a high pretreatment GNRI level; and (4) O (outcomes): studies that reported the prognostic role of GNRI for any survival outcome, including overall survival (OS), progression-free survival, and cancer-specific survival (CSS). A cut-off value to divide patients into low and high GNRI groups was identified for (2) and (3). The hazard ratios (HRs) and corresponding 95% confidence intervals (CIs) for (4) were either directly reported by the studies or could be calculated using the data provided.

The exclusion criteria were as follows: (1) meeting abstracts, case reports, letters, reviews, and comments; (2) duplicate studies; and (3) animal experiments.

### Data extraction and quality assessment

Two investigators (LH and YL) independently extracted the information from eligible studies, and all disagreements were resolved by consensus with a third investigator (FZ). The following data were extracted from each included study: name of the first author, year of publication, country, recruitment period, sample size, tumor stage, treatment, age, sex, follow-up, cut-off value of the GNRI, HR analysis type, survival endpoints, and HRs with 95% CIs for survival outcomes. The quality of all included studies was systematically evaluated using the Newcastle-Ottawa Scale (NOS) (24). The NOS scores ranged from 0 to 9. Studies with the NOS scores ≥6 were considered high-quality research.

### Statistical analyses

All statistical analyses were performed using Stata version 12.0 (Stata Corporation, College Station, Texas, USA). The pooled HRs and 95% CIs were used to estimate the prognostic value of the GNRI in GC. Heterogeneity among all included studies was analyzed using the chi-squared test and quantitatively assessed using the *I*^2^ value. *I*^2^ > 50% or *P* for heterogeneity <0.10 indicates significant heterogeneity, and a random-effects model was applied for this event. Otherwise, a fixed effects model was used. Subgroup analyses were performed to evaluate the prognostic effect of the GNRI in various subgroups. Odds ratios (ORs) and 95% CIs were used to assess the correlation between the GNRI and clinicopathological characteristics of GC. Begg's test and funnel plots were used to assess potential publication bias. Statistical significance was set at *P* < 0.05.

### Ethics statement

Ethical review and approval were waived because this study summarized the published literature.

## Results

### Study retrieval

**Figure 1:**
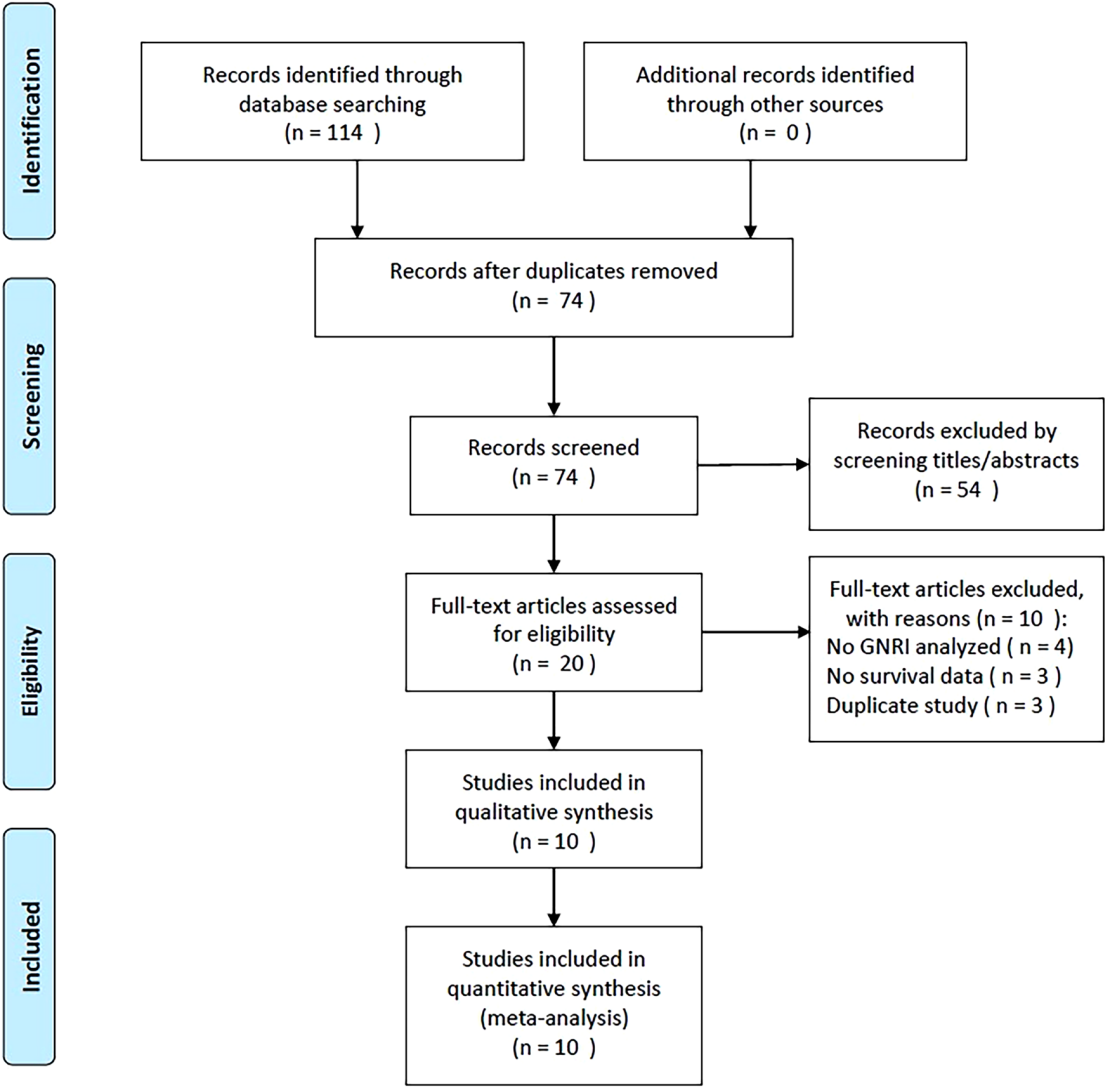
Flow chart of literature retrieval and research selection.

A flowchart of the study selection process is presented in Figure 1. During the initial literature retrieval, we identified 114 records, of which 74 remained after the removal of duplicate records. After screening titles and abstracts, 54 studies were further excluded, and the remaining 20 were evaluated by full-text examination. Subsequently, ten studies were excluded for the following reasons: four did not include the GNRI in the analyses, three did not provide survival data, and three recruited overlapping patients. Ultimately, 10 studies recruiting 5,834 patients (13–22) with GC were included in this meta-analysis (Figure 1, Table 1).

**Table 1:** Baseline characteristics of included studies in the present meta-analysis.

Study	Year	Country	Study duration	Sample size	Tumor stage	Age (year) median (range)	Gender (M/F)	Treatment	Cut-off value	Follow-up (month) median (range)	Survival outcome	HR type	NOS score
Hirahara, N.	2020	Japan	2010–2017	297	I–III	74 (65–89)	205/92	Surgery	94.8	36.0 (2.8–96.5)	OS, CSS	Multivariate	8
Furuke, H.	2021	Japan	2008–2016	795	I–III	68 (29–89)	534/261	Surgery	92	1–60	OS, CSS	Multivariate	7
Shimada, T.	2021	Japan	2004–2017	106	EGC	76	83/23	ESD	92	89	CSS	Multivariate	7
Sugawara, K.	2021	Japan	2001–2014	1,166	I–III	63 (25–91)	816/350	Surgery	98	82.2	OS, CSS	Multivariate	8
An, S.	2022	Korea	2006–2017	450	I–III	60 (26–92)	301/149	Surgery	92	72	OS	Univariate	7
Hisada, H.	2022	Japan	2009–2019	767	EGC	75 (65–95)	559/208	ESD	92	56.1 (0.6–147.6)	OS	Multivariate	8
Matsunaga, T.	2022	Japan	2005–2015	497	I–III	80.6	325/164	Surgery	97	1–60	OS, CSS	Multivariate	8
Toya, Y.	2022	Japan	2002–2017	740	EGC	86	469/271	ESD	96	1–60	OS	Univariate	7
Tsuchiya, N.	2022	Japan	2002–2018	186	I–III	82 (80–93)	128/58	Surgery	98	1–60	OS	Univariate	6
Yoshikawa, T.	2022	Japan	2006–2020	830	EGC	35–96	615/215	ESD	92	1–166	OS	Multivariate	8

M, male; F, female; EGC, early gastric cancer; ESD, endoscopic submucosal dissection; OS, overall survival; CSS, cancer-specific survival; HR, hazard ratio; NOS, Newcastle-Ottawa Scale.

### Characteristics of included studies

Basic characteristics of the included studies are summarized in Table 1. All 10 studies (13–22) were of a retrospective design. Nine studies were conducted in Japan (13–16, 18–22) and one in Korea (17). The total sample size was 5,834, ranging from 106 to 1,166. Six studies recruited patients with stages I-III GC (13, 14, 16, 17, 19, 21), and four studies enrolled patients with early GC (15, 18, 20, 22). Five studies selected 92 as the cut-off value of the GNRI (14, 15, 17, 18, 22), two selected 98 (16, 21), and three selected 94.8 (13), 96 (20), and 97 (19) as their respective GNRI cut-off values. Nine studies reported the prognostic value of the GNRI for OS (13, 14, 16–22), and five studies presented HRs and 95% CIs for CSS (13–16, 19). Seven studies provided HRs and 95% CIs using multivariate analysis (13–16, 18, 19, 22), whereas three studies used univariate analysis (17, 20, 21). The NOS scores of the included studies ranged from 6 to 8, with a median value of 7.5, suggesting that all the included studies were of high quality.

### GNRI and OS

**Figure 2:**
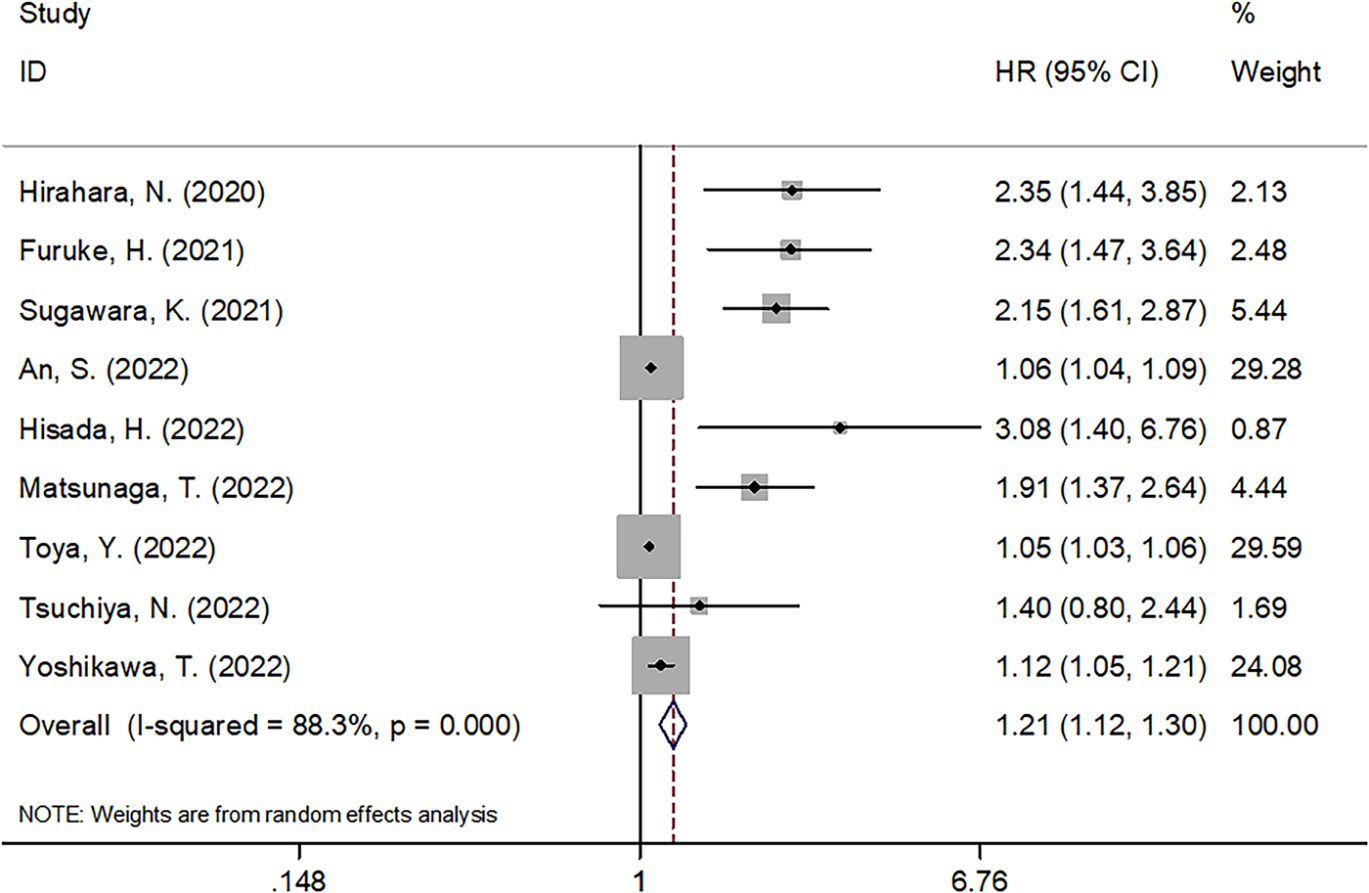
Forrest plots of the correlation between GNRI and OS in GC patients.

A total of nine studies with 5,728 patients (13, 14, 16–22) demonstrated the prognostic role of the GNRI for OS in GC. A random-effects model was applied because of the significant heterogeneity (*I*^2^ = 88.3%, Ph < 0.001). As shown in Figure 2 and Table 2, pooled HR = 1.21, 95% CI = 1.12–1.30, and *P* < 0.001, which suggests that a lower GNRI is a significant prognostic biomarker for patients with GC. Subgroup analysis was conducted through stratification of diverse factors, including sample size, country, treatment, cut-off value, and HR type. As shown in Table 2, the combined results suggest that a decreased GNRI remains a significant prognostic indicator for worse OS, irrespective of sample size, country, cut-off value, and HR type (all *P* < 0.05).

**Table 2:** Subgroup analysis of prognostic value of GNRI for OS and CSS in patients with gastric cancer.

Subgroup	No. of studies	No. of patients	HR (95% CI)	P	Effects model	Heterogeneity I^2^ (%) Ph
OS							
Overall	9	5,728	1.21 (1.12–1.30)	<0.001	Random	88.3	<0.001
Sample size							
≤500	4	1,430	1.56 (1.02–2.41)	0.042	Random	86.9	<0.001
>500	5	4,298	1.40 (1.17–1.67)	<0.001	Random	91.1	<0.001
Country							
Japan	8	5,278	1.55 (1.31–1.84)	<0.001	Random	89.8	<0.001
Korea	1	450	1.06 (1.04–1.09)	<0.001	-	-	-
Treatment							
Surgery	6	3,391	1.77 (1.20–2.62)	0.004	Random	91.2	<0.001
ESD	3	2,337	1.10 (1.00–1.23)	0.062	Random	80.8	0.006
Cut-off value							
≤92	4	2,842	1.20 (1.04–1.38)	0.012	Random	85.6	<0.001
>92	5	2,886	1.68 (1.10–2.55)	0.016	Random	91.5	<0.001
HR type							
Multivariate	6	4,352	1.97 (1.34–2.90)	0.001	Random	89.5	<0.001
Univariate	3	1,376	1.06 (1.04–1.07)	<0.001	Fixed	0	0.481
CSS							
Overall	5	2,861	2.21 (1.75–2.80)	<0.001	Fixed	0	0.526
Sample size							
≤500	3	900	1.76 (1.15–2.70)	0.009	Fixed	0	0.996
>500	2	1,961	2.45 (1.84–3.25)	<0.001	Fixed	37.9	0.204
Treatment							
Surgery	4	2,755	2.22 (1.75–2.82)	<0.001	Fixed	3.6	0.375
ESD	1	106	1.60 (0.17–15.03)	0.681	-	-	-
Cut-off value							
≤92	2	901	1.99 (1.33–3.00)	0.001	Fixed	0	0.845
>92	3	1,960	2.33 (1.75–3.11)	<0.001	Fixed	28.0	0.250

GNRI, geriatric nutritional risk index; OS, overall survival; CSS, cancer-specific survival; HR, hazard ratio; ESD, endoscopic submucosal dissection.

### GNRI and CSS

**Figure 3:**
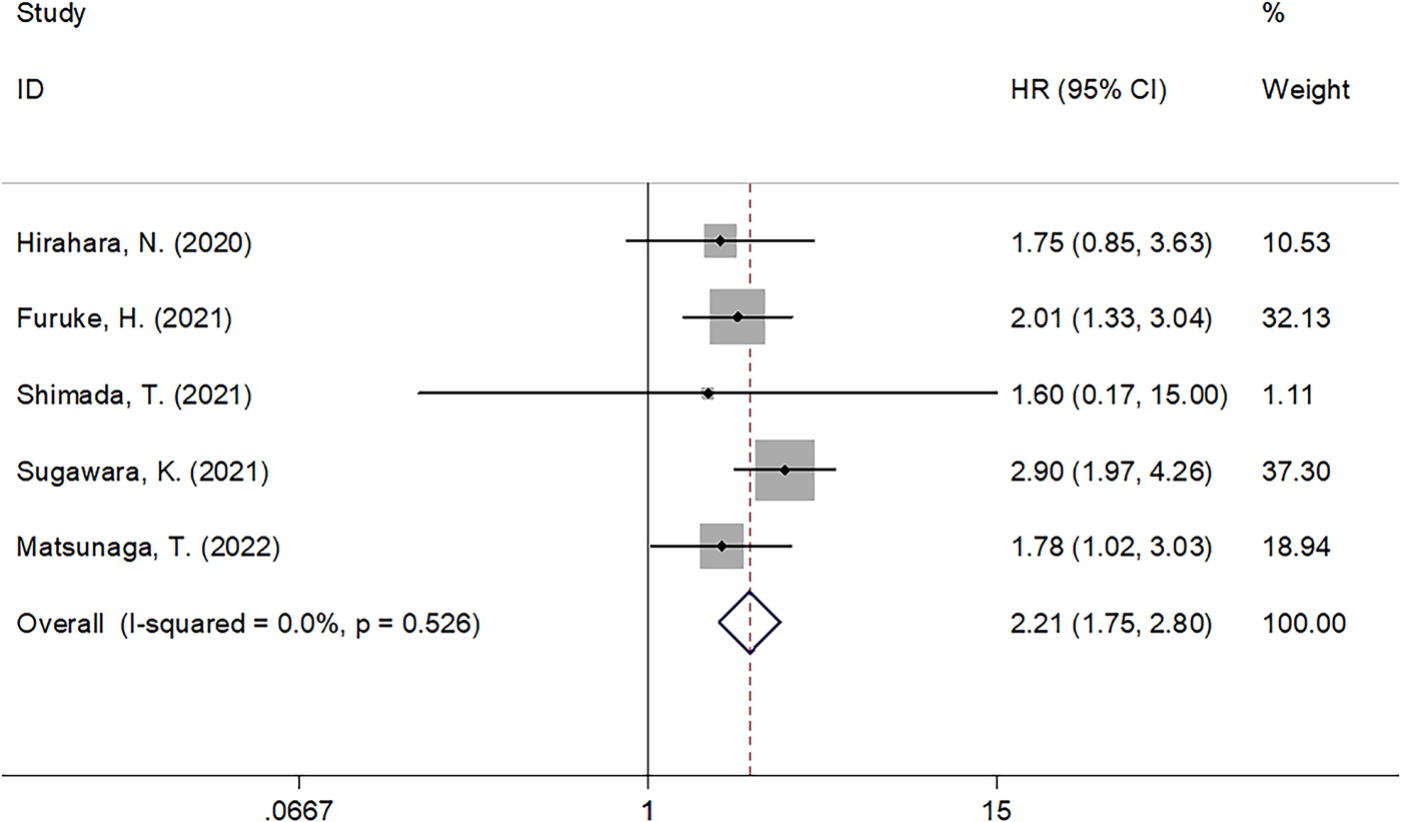
Forrest plots of the correlation between GNRI and CSS in GC patients.

Five studies comprising 2,861 patients (13–16, 19) investigated the prognostic efficiency of the GNRI for CSS in GC. There was no significant heterogeneity (*I*^2^ = 0, Ph = 0.526), and a fixed-effects model was used. As shown in Table 2 and Figure 3, our results indicate that a lower GNRI is a significant prognostic marker for poor CSS in GC (HR = 2.21, 95% CI = 1.75–2.80, *P* < 0.001). Subgroup analysis demonstrated that the prognostic role of the GNRI for CSS was not affected by sample size or cut-off value (all *P* < 0.05) (Table 2).

### Relationship between GNRI and clinicopathological factors

**Figure 4:**
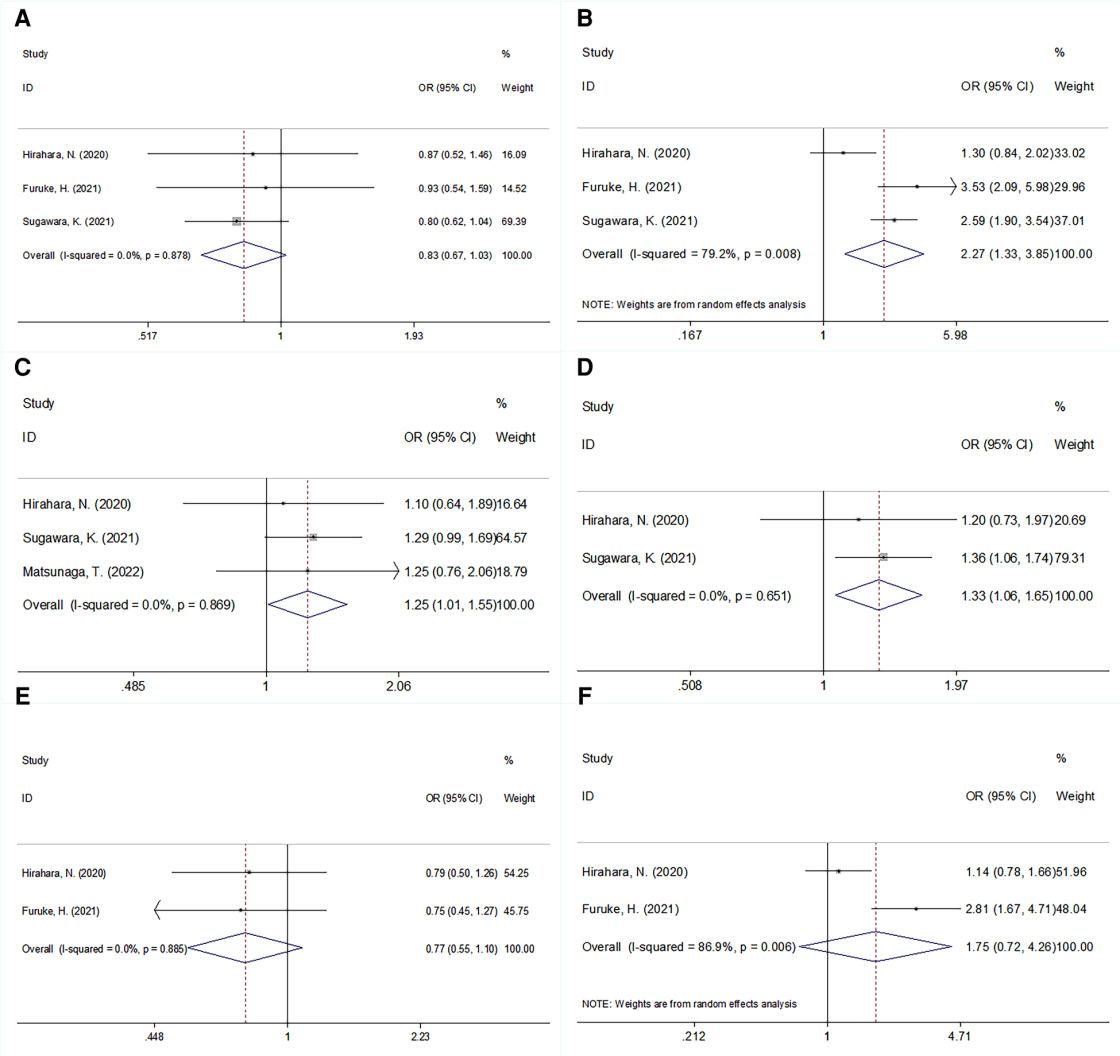
Forrest plots of the relationship between GNRI and clinicopathological features in patients with GC. (**A**) Gender (male vs. female); (**B**) pathological stage (III vs. I–II); (**C**) adjuvant chemotherapy (presence vs. absence); (**D**) tumor location (lower vs. upper + middle); (**E**) tumor differentiation (poor vs. well/moderate); and (**F**) lymph node metastasis (N+ vs. N0).

The correlation between the GNRI and multiple clinicopathological features was explored in four studies that included 2,755 patients (13–16, 19). As shown in Figure 4 and Table 3, our pooled data illustrates that a low GNRI is significantly associated with advanced pathological stage (OR = 2.27, 95% CI = 1.33–3.85, *P* = 0.003), presence of adjuvant chemotherapy (OR = 1.25, 95% CI = 1.01–1.55, *P* = 0.040), and tumor location in the lower stomach (OR = 1.33, 95% CI = 1.06–1.65, *P* = 0.012). However, there was no significant association between the GNRI and sex (OR = 0.83, 95% CI = 0.67–1.03, *P* = 0.087), tumor differentiation (OR = 0.77, 95% CI = 0.55–1.10, *P* = 0.148), or lymph node metastasis (OR = 1.75, 95% CI = 0.72–4.26, *P* = 0.214; Figure 4, Table 3) in patients with GC.

**Table 3:** The association between GNRI and clinicopathological features in patients with gastric cancer.

Factors	No. of studies	No. of patients	OR (95% CI)	*P*	Effects model	Heterogeneity	
						*I*^2^ (%)	Ph
Gender (male vs. female)	3	2,258	0.83 (0.67–1.03)	0.087	Fixed	0	0.878
Pathological stage (III vs. I–II)	3	2,258	2.27 (1.33–3.85)	0.003	Random	79.2	0.008
Adjuvant chemotherapy (presence vs. absence)	3	1,960	1.25 (1.01–1.55)	0.040	Fixed	0	0.869
Tumor location (lower vs. upper + middle)	2	1,463	1.33 (1.06–1.65)	0.012	Fixed	0	0.651
Tumor differentiation (poor vs. well/moderate)	2	1,092	0.77 (0.55–1.10)	0.148	Fixed	0	0.885
LN metastasis (N+ vs. N0)	2	1,092	1.75 (0.72–4.26)	0.214	Random	86.9	0.006

GNRI, geriatric nutritional risk index; OR, odds ratio; LN, lymph node.

### Publication bias

**Figure 5:**
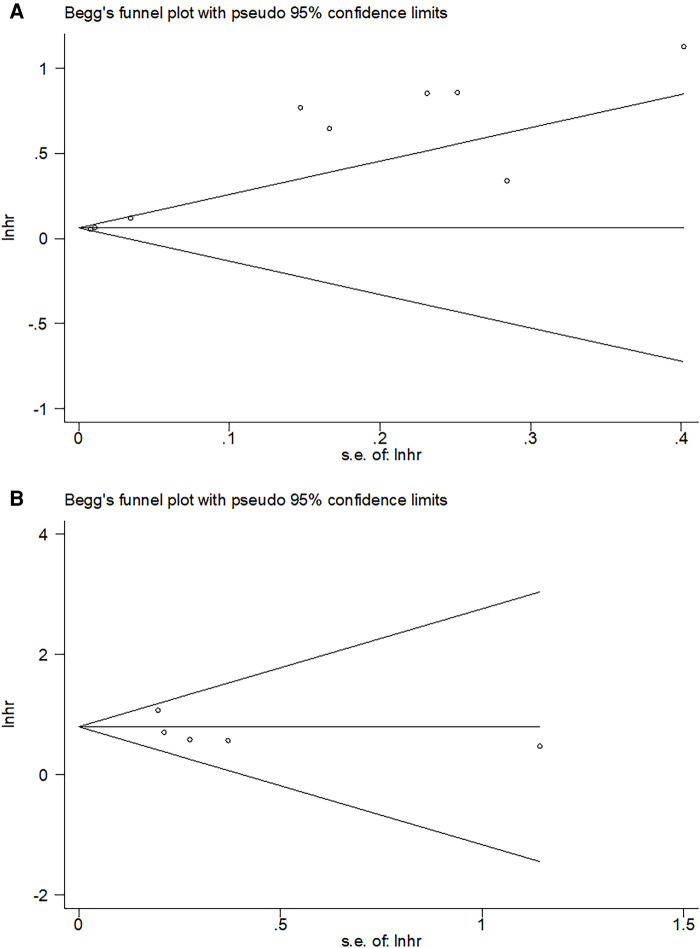
Begg's funnel plot. (**A**) OS, *P* = 0.602; (**B**) CSS, *P* = 0.806.

Potential publication bias was detected using funnel plots and Begg's test. As shown in Figure 5, the shape of the funnel diagram was symmetrical. Moreover, the results of Begg's test (*P* = 0.602 for OS and *P* = 0.806 for CSS) also demonstrated no significant publication bias in this meta-analysis.

## Discussion

Previous studies have explored the prognostic significance of the GNRI in patients (13–22), with inconsistent results. In the current meta-analysis, we retrieved data from 10 studies comprising 5,834 cases to systemically shed light on this issue. Our results demonstrated that a lower GNRI is a significant prognostic indicator of worse OS and CSS in patients with GC. The subgroup analysis confirmed the robustness of the results. Furthermore, we also found a significant association between the GNRI and advanced pathological stage, presence of adjuvant chemotherapy, and tumor location in the lower stomach in patients with GC. Taken together, our meta-analysis showed that the pretreatment GNRI is a reliable and readily available prognostic marker for survival outcomes in GC. In addition, patients with GC who have a lower GNRI may have an advanced pathological stage and should be treated with adjuvant chemotherapy following surgical resection.

The GNRI is an objective and simple predictive tool for nutritional status. The underlying mechanism by which a low GNRI results in worse prognosis than a high GNRI among patients with GC is unclear. The GNRI is calculated from albumin, height, and body weight, which are generally measured on admission for most patients. Therefore, the acquisition of the GNRI is easy and cost-effective. A low GNRI can be the result of low serum albumin levels and being underweight. The association between the GNRI and poor prognosis in GC can be explained as follows. First, albumin is the most commonly used indicator in the clinical evaluation of patients’ nutritional status (25). Malnutrition is closely associated with impaired immune function, which weakens host antitumor immunity. Hypoalbuminemia is regarded as an indicator of chronic malnutrition and has been proven to be associated with poor long-term prognosis in hospitalized patients, including those with GC (26, 27). Second, weight loss is expected with negative cell-regulating mechanisms of cancer or in patients with aggressive cancers. Lower body weight and body mass index are well-established prognostic factors in patients with various types of cancer (28, 29). Taken together, a low GNRI is a stable prognostic indicator for patients with GC.

The strengths of this meta-analysis are the following. First, the sample size in this meta-analysis was large. A total of 5,834 patients were included, representing a relatively comprehensive patient population. Second, the publication years of all the included studies were in the last 3 years (2020–2022), and more than half of the studies were published in 2022. Therefore, this meta-analysis considers the most recent and updated data on the association between the GNRI and survival in GC. Third, all the included studies were published in English, so their availability is good. Our meta-analysis showed some hematological parameters that are promising prognostic factors in patients with cancer. These indexes are simple and easily accessible, with no additional costs to or examination of patients. Additional research efforts should be devoted to hematological prognostic factors. Furthermore, this meta-analysis has implications for clinical practice. During treatment of patients with GC who have a low GNRI, attention should be paid to their nutritional status. Improved nutrition in patients with a low GNRI may prevent poor prognosis.

Subgroup analyses showed that a low GNRI remained a significant prognostic factor for worse OS in GC in subgroups of sample size, country, cut-off value, and HR type (all *P* < 0.05; Table 2). However, a decreased GNRI predicted OS in patients with GC undergoing surgery (HR = 1.77, 95% CI = 1.20–2.62, *P* = 0.004), but not in those undergoing endoscopic submucosal dissection (ESD) (HR = 1.10, 95% CI = 1.00–1.23, *P* = 0.062) (Table 2). Similar results were also found for CSS (Table 2). This result is interesting and can be explained as follows. First, patients with GC undergoing ESD must meet the gastric cancer treatment guidelines (30) and are usually diagnosed with early GC. In contrast, patients undergoing surgery are in the early and advanced stages and typically undergo adjuvant chemotherapy (30). Second, ESD is less invasive than surgery. Therefore, malnutrition is less prevalent in patients with GC undergoing ESD than in those undergoing surgery. Moreover, the GNRI is not a prognostic marker for patients with GC undergoing ESD. The GNRI can also be applied in combination with other nutritional indexes, including the prognostic nutritional index, controlling nutritional status score, albumin-to-globulin ratio, and C-reactive protein to albumin ratio, to improve the prognostic efficiency for GC. Nomograms based on these parameters can also be explored in future studies.

Many meta-analyses have recently reported the prognostic significance of the GNRI in a variety of solid tumors (31–35). A recent meta-analysis including 3,981 patients showed that the pretreatment GNRI was significantly associated with prognosis in patients with esophageal cancer, and a lower GNRI predicted a worse survival rate (32). Another meta-analysis indicated that patients with lung cancer with a lower pretreatment GNRI had inferior prognoses on the basis of data from 2,012 patients (36). Zhao et al. reported in their meta-analysis that the GNRI at baseline could be an independent predictor of poor survival outcomes in patients with colorectal cancer (31). In this meta-analysis, we observed a significant prognostic role of GNRI in GC, which was in line with the findings for other types of cancer, suggesting the general prognostic impact of GNRI on solid tumors.

This meta-analysis had some limitations. First, all the included studies had a retrospective design. Therefore, a selection bias may exist. Second, all the included studies were conducted in East Asia, although we did not restrict the geographical regions of the eligible studies. However, the publication language of all the studies was English. This may be due to Japan's efforts to prevent and treat gastric cancer (37–39). Third, the cutoff values of the GNRI ranged from 92 to 98, which may have introduced heterogeneity in this meta-analysis. Fourth, the heterogeneity exists and is significant in several analysis groups, including in OS, pathological stage, and lymph node metastasis (Tables 2, 3). To address this, we used a random effects model in these groups. Thus, large multicenter prospective trials are needed to validate the prognostic role of the GNRI in patients with GC.

In summary, our meta-analysis identified that the pretreatment GNRI level was a significant prognostic factor for patients with GC. A low GNRI is associated with worse OS and inferior CSS in patients with GC.

## Data Availability

The original contributions presented in the study are included in the article/**Supplementary Material**, further inquiries can be directed to the corresponding author.

## Supplementary material

Supplementary Table S1 The Prisma
